# Aerosol delivery of synthetic lung surfactant

**DOI:** 10.7717/peerj.403

**Published:** 2014-05-27

**Authors:** Frans J. Walther, José M. Hernández-Juviel, Alan J. Waring

**Affiliations:** 1 Department of Pediatrics, Division of Medical Genetics, Los Angeles Biomedical Research Institute, Harbor-UCLA Medical Center, Torrance, CA, USA; 2 Department of Pediatrics, David Geffen School of Medicine, University of California, LA, USA; 3 Department of Medicine, Division of Molecular Medicine, Los Angeles Biomedical Research Institute, Harbor-UCLA Medical Center, Harbor-UCLA Medical Center, Torrance, CA, USA; 4 Department of Medicine, David Geffen School of Medicine, University of California, LA, USA; 5 Department of Physiology & Biophysics, School of Medicine, University of California, Irvine, CA, USA

**Keywords:** Aerosol delivery, Synthetic lung surfactant, Captive bubble surfactometry, Surfactant-deficient rabbits, Lung function, Mechanical ventilation, Surfactant protein B, Surfactant protein C, Nasal continuous positive airway pressure

## Abstract

**Background.** Nasal continuous positive airway pressure (nCPAP) is a widely accepted technique of non-invasive respiratory support in premature infants with respiratory distress syndrome due to lack of lung surfactant. If this approach fails, the next step is often intubation, mechanical ventilation (MV) and intratracheal instillation of clinical lung surfactant.

**Objective.** To investigate whether aerosol delivery of advanced synthetic lung surfactant, consisting of peptide mimics of surfactant proteins B and C (SP-B and SP-C) and synthetic lipids, during nCPAP improves lung function in surfactant-deficient rabbits.

**Methods.** Experimental synthetic lung surfactants were produced by formulating 3% Super Mini-B peptide (SMB surfactant), a highly surface active SP-B mimic, and a combination of 1.5% SMB and 1.5% of the SP-C mimic SP-Css ion-lock 1 (BC surfactant), with a synthetic lipid mixture. After testing aerosol generation using a vibrating membrane nebulizer and aerosol conditioning (particle size, surfactant composition and surface activity), we investigated the effects of aerosol delivery of synthetic SMB and BC surfactant preparations on oxygenation and lung compliance in saline-lavaged, surfactant-deficient rabbits, supported with either nCPAP or MV.

**Results.** Particle size distribution of the surfactant aerosols was within the 1–3 µm distribution range and surfactant activity was not affected by aerosolization. At a dose equivalent to clinical surfactant therapy in premature infants (100 mg/kg), aerosol delivery of both synthetic surfactant preparations led to a quick and clinically relevant improvement in oxygenation and lung compliance in the rabbits. Lung function recovered to a greater extent in rabbits supported with MV than with nCPAP. BC surfactant outperformed SMB surfactant in improving lung function and was associated with higher phospholipid values in bronchoalveolar lavage fluid; these findings were irrespective of the type of ventilatory support (nCPAP or MV) used.

**Conclusions.** Aerosol delivery of synthetic lung surfactant with a combination of highly active second generation SP-B and SP-C mimics was effective as a therapeutic approach towards relieving surfactant deficiency in spontaneously breathing rabbits supported with nCPAP. To obtain similar results with nCPAP as with intratracheal instillation, higher dosage of synthetic surfactant and reduction of its retention by the delivery circuit will be needed to increase the lung dose.

## Introduction

Approximately 7% of all infants are born prematurely and half of them develop respiratory distress syndrome (RDS) due to lung immaturity and lack of lung surfactant, a mixture of (phospho)lipids and (surfactant) proteins that reduces alveolar surface tension in the lungs to extremely low values and prevents alveolar collapse during expiration. Nasal continuous positive airway pressure (nCPAP), which provides noninvasive respiratory support by alveolar recruitment and improving functional residual capacity during spontaneous breathing, has revolutionized the respiratory management of premature infants because it not only provides lung protection from the first breath onwards, but is also applicable in both high and low technical settings. In case nCPAP fails, the next step is usually intubation, mechanical ventilation, intratracheal surfactant instillation, and, if possible, rapid extubation and return to nCPAP (the so called “InSurE” approach) (Herting, 2013). However, endotracheal intubation and surfactant instillation is invasive, requires advanced skills and equipment, and may lead to complications, such as bradycardia, hypotension, and desaturations due to tube obstruction and/or surfactant reflux. Many neonatal intensive care units have switched to alternative, less invasive techniques whereby infants receive surfactant treatment during spontaneous breathing via a thin catheter inserted into the trachea by laryngoscopy (Göpel et al., 2011; Dargaville et al., 2013). Delivery of exogenous surfactant as an aerosol during spontaneous breathing without manipulation in the oropharynx and laryngeal airways would not only resolve the instillation problems, but also open up the possibility to combine nCPAP and surfactant treatment in low technical settings.

The combination of nCPAP and aerosolized surfactant has been explored in small pilot studies in premature infants with clinical (animal-derived) lung surfactants and first generation synthetic surfactants (Exosurf Neonatal^®^ and Surfaxin^®^) (Jorch et al., 1997 [Alveofact^®^]; Arroe et al., 1998 [Exosurf Neonatal^®^]; Berggren et al., 2000 [Curosurf^®^]; Finer et al., 2010 [Surfaxin^®^]), but these studies did not provide good data on clinical efficacy, aerosol generation and nCPAP, and particle size in lung deposition (Pillow & Minocchieri, 2012). As a result, a recent Cochrane Systematic Review concluded that there are insufficient data to support or refute the use of nebulized surfactant in clinical practice (Abdel-Latif & Osborn, 2012). However, the emergence of advanced synthetic surfactants with SP-B and SP-C mimics (Curstedt, Calkovska & Johansson, 2013) and the expanding use of noninvasive ventilation in premature infants with RDS have raised the demand for synthetic surfactant formulated for use as an aerosol.

Our group has developed various highly active surfactant protein B and C (SP-B and SP-C) mimics, the two core surfactant proteins that drive the reduction in alveolar surface tension at the monolayer covering the alveolar epithelium (Curstedt, Calkovska & Johansson, 2013). Super Mini-B (SMB) is a 41-residue SP-B mimic based on the primary sequence, tertiary folding and quaternary association properties of the 79-residue native protein (Walther et al., 2010). SMB reproduces the topology of the N- and C-terminal domains of SP-B as it contains the *α*-helical residues 1–25 and 63–78 of SP-B joined with a custom beta-turn—PKGG—and has shown excellent surface activity as single surfactant peptide in various lipid mixtures (Walther et al., 2010). SP-Css ion-lock 1 is a 34-residue SP-C mimic that simulates the structural and functional properties of the native SP-C sequence without palmitoyls by replacing the two cysteines in the N-terminal with serines and by swapping two valines in the *α*-helix with a single salt-bridge (with Glu^−^-20 and Lys^+^-24 insertions) in the mid-section of hydrophobic C-terminal region to stabilize the *α*-helix. FTIR spectroscopy has demonstrated that SP-Css ion lock 1 closely simulates the high *α*-helicity and membrane topography of native dipalmitoylated SP-C and *in vitro* and *in vivo* studies have confirmed excellent surface activity (RH Notter, AJ Waring, FJ Walther, LM Gordon, and Z Wang, 2012, unpublished data). Both synthetic surfactant peptides are highly surface active in a simple lipid mixture consisting of dipalmitoyl-phosphatidylcholine (DPPC), palmitoyl-oleoyl-phosphatidylcholine (POPC) and palmitoyl-oleoyl-phosphatidylglycerol (POPG) at a weight ratio of 5:3:2 (Walther et al., 2005).

In this study, we first tested aerosol generation and conditioning (particle size, surfactant composition and surface activity) and clinical efficacy in animal experiments of SMB surfactant, and then repeated some of the measures to investigate the potential additive value of SP-Css ion-lock 1 peptide.

## Materials & Methods

Peptide synthesis reagents were purchased from Applied Biosystems (Foster City, CA), high performance liquid chromatography solvents from Fisher Chemical Co. (Pittsburgh, PA), and all other chemicals from Sigma Chemical Co. (St. Louis, MO) and Aldrich Chemical Co. (Milwaukee, WI). DPPC, POPC and POPG were from Avanti Polar Lipids (Alabaster, AL). The clinical surfactant Infasurf^®^ (Calfactant), a bovine lung lavage extract, was a generous gift from Ony Inc. (Amherst, NY). Young adult New Zealand White rabbits, weighing 1.0–1.3 kg, were obtained from I.F.P.S. (Norco, CA).

### Synthesis and characterization of surfactant peptides

SMB peptide (41 residues, linear sequence NH_2_-FPIPLPYCWLCRALIKRIQAMIPKGGRMLPQLVCRLVLRCS-COOH) (Walther et al., 2010) and SP-Css ion lock 1 peptide (34 residues, linear sequence NH_2_-GIPSSPVHLKRLLIVVVVVELIVKVIVGALLMGL-COOH) (RH Notter, AJ Waring, FJ Walther, LM Gordon, and Z Wang, 2012, unpublished data) were synthesized on a Symphony Multiple Peptide Synthesizer (Protein Technologies, Tucson, AZ) with standard Fmoc chemistry. Crude peptides were purified by reverse phase high performance liquid chromatography, molecular weights were verified by MALDI-TOF, alpha-helicity was determined by Fourier Transform InfraRed spectroscopy, and disulfide connectivity was confirmed by mass spectroscopy of enzyme-digested fragments (trypsin and chymotrypsin digestion) (Walther et al., 2010).

### Surfactant preparations

The two experimental synthetic surfactant mixtures to treat surfactant deficiency were 3% Super Mini-B (SMB) and 1.5% SMB + 1.5% SP-Css ion lock 1 (BC) formulated with a synthetic phospholipid mixture, consisting of DPPC:POPC:POPG 5:3:2 (weight ratio) at a concentration of 35 mg phospholipids/ml. The composition of the synthetic phospholipid mixture was based on the lipid composition of native lung surfactant (Walther et al., 2005). In recent studies on SP-B and SP-C mimics we used Infasurf^®^ (a bovine lung lavage extract containing both native SP-B and SP-C) and synthetic lipids alone as positive and negative control in ventilated, lavaged rabbits (Walther et al., 2010; Walther et al., 2014) and we therefore only treated a small number of lavaged rabbits, supported with nasal CPAP, with Infasurf as a control group.

### Nebulizer

The Aeroneb Pro^®^ nebulizer (Aerogen Inc., Mountain View, CA) (Dubus et al., 2005) was used to aerosolize liquid formulations without affecting the compound’s composition and concentration. This small volume (10 ml max) median diameter nebulizer delivers a low velocity aerosol with a particle size <3.0 µm with a flow rate >0.1 ml/min, does not need a gas source and is FDA-approved for human use. Finer et al. (2010) have shown that this nebulizer delivers particles with a mass median aerodynamic diameter (MMAD) of 1.9 ± 0.3 µm. The nebulizer was placed directly below the “Y” connector, i.e., below the circuit and above the nasal prongs as this position, in combination with nCPAP, results in higher dose deposits (Mazela & Polin, 2011). Aerosol characteristics were measured with laser diffraction (Malvern Instruments, Westborough, MA) and output from the nebulizer was determined from mass loss at room temperature. Mass loss was calculated after measuring the mass of the aerosol collected from the tips of the nasopharyngeal prongs.

### Captive bubble surfactometry

Adsorption and dynamic surface tension lowering ability of all surfactant preparations were measured with a captive bubble surfactometer at physiological cycling rate, area compression, temperature, and humidity (Walther et al., 2010). We routinely analyze surfactant samples of 1 µl (35 mg phospholipids/ml) in the captive bubble surfactometer and perform all measurements in quadruplicate. Surface activity of all surfactant preparations was measured prior to their use in animal experiments and repeated using aerosolized material.

### Animal experiments

Animal experiments were performed using a protocol (research project 20645-01) approved by the Animal Care and Use Committee at the Los Angeles Biomedical Research Institute at Harbor-UCLA Medical Center. All procedures and anesthesia were in accordance with the American Veterinary Medical Association (AMVA) Guidelines.

Twenty-four young adult rabbits (body weight of 1.0–1.3 kg) received anesthesia with 50 mg/kg of ketamine and 5 mg/kg of acepromazine intramuscularly prior to placement of a venous line via a marginal ear vein. After intravenous administration of 1 mg/kg of diazepam and 0.2 mg/kg of propofol, a small incision was made in the skin of the anterior neck for placement of a carotid arterial line to monitor heart rate and blood pressure. Rabbits were intubated orally and stabilized on a standard ventilator (tidal volume targeted to 6 ml/kg, a positive inspiratory pressure [PIP] of 12 cm H_2_O, a positive end-expiratory pressure [PEEP] of 2 cm H_2_O, inspiratory time 0.5 s, ventilator rate 40/min [and adjusted to keep PaCO_2_ 35–45 mm Hg], 100% oxygen). Airway flow and pressures and tidal volume were monitored continuously with a pneumotachograph connected to the endotracheal tube and a pneumotach system (Hans Rudolph Inc., Kansas City, MO). Body temperature was monitored continuously with a rectal temperature probe and maintained with a heating pad. After instrumentation, arterial pH and blood gases were measured. If the PaO_2_ was >500 mm Hg at a PIP <15 cm H_2_O, the rabbit underwent repeated bronchoalveolar lavages (BAL), i.e., ∼3 lung lavages with 30 ml of prewarmed 0.9% NaCl with a short recovery period in between, until PaO_2_ values <150 mm Hg were reached. At this point half of the rabbits were assigned to continue MV while paralyzed (vecuronium 0.1 mg/kg intravenously) and the other half was weaned to nCPAP, using custom-made nasopharyngeal prongs, after spontaneous breathing was established. Mechanical ventilation was continued with a tidal volume targeted to 6 ml/kg (PIP ∼22 cm H_2_O with a range of 20–25 cm H_2_O based on the tidal volume measures, a PEEP of 3 cm H_2_O, inspiratory time 0.5 s, ventilator rate 40/min, 100% oxygen). Nasal CPAP was delivered via the ventilator with a standard PEEP of 5–6 cm H_2_O. After respiratory failure was re-confirmed with a repeat blood gas, 100 mg/kg of experimental surfactant or Infasurf was loaded into the chamber of the vibrating membrane nebulizer (Aeroneb Pro^®^, Aerogen Inc., Mountain View, CA) and the aerosol was delivered over a period of 15 min. Arterial pH and blood gases were repeated at 15 min intervals after surfactant aerosolization. Animals were sacrificed 120 min after surfactant administration with an intravenous overdose of pentobarbital (200 mg/kg), a standardized BAL was done (3 lavages of 30 ml each), and the lungs were checked for air leaks, bleedings and atelectasis. End-points were arterial pH and blood gases, dynamic compliance, and lung lavage proteins and lipids.

### Protein and phospholipid measurements of BAL

Protein and phospholipid measurements of BAL fluid were performed in samples collected during the first standard lung lavage to induce surfactant deficiency and the first of three portmortem lung lavages. Protein was measured using the Lowry assay with human albumin as a standard (Lowry et al., 1951). Phospholipids were measured by extracting BAL samples in chloroform:methanol 2:1 v:v (1 ml of lavage + 4 ml chloroform:methanol) and applying the extract to the attenuated total reflection Fourier-transform infrared spectroscopy plate and drying it before taking a spectrum (Goormaghtigh, Cabiaux & Ruysschaert, 1990).

### Data analysis

Data are expressed as means ± standard error (SEM). Statistical differences were estimated using *t*-tests and analyses of variance (ANOVA) using IBM SPSS statistics software version 22. Student’s *t*-test was used for comparisons versus control values. Between groups comparisons at various time-points were done by one-way ANOVA and time courses were analyzed with one-way repeated measure ANOVA. A *p* value <0.05 was considered to indicate a significant difference.

## Results

### Aerosol and surfactant characteristics

The MMAD of both synthetic surfactant preparations was between 1 and 3 µ and within the range previously established for the Aeroneb Pro nebulizer, i.e., <3 µm average MMAD (Finer et al., 2010). Nebulizer residue in the holding chamber was lowest for SMB surfactant and synthetic lipids alone, intermediate for BC surfactant and highest for Infasurf (Table 1).

**Table 1:** Nebulizer residue and delivery at the tip of the nasopharyngeal prongs of surfactant aerosol. The delivery of the experimental surfactant preparations and synthetic lipids at the tip of the nasopharyngeal prongs was calculated as the percentage of the surfactant dose placed in the holding chamber of the nebulizer that was recovered at the distal end of the nasopharyngeal prongs. Data are expressed as mean ± SD % of *n* = 4.

Surfactant	Nebulizer residue	Delivery at the tip of the nasopharyngeal prongs
Synthetic lipids alone	10.5 ± 3.8 %	50.5 ± 15.9 %
SMB surfactant	4.7 ± 2.1 %	55.2 ± 14.2 %
BC surfactant	24.4 ± 5.3 %	39.6 ± 3.2 %
Infasurf	36.2 ± 7.0 %	32.6 ± 6.3 %

Aerosol delivery measured at the tip of the nasopharyngeal prongs was highest for SMB surfactant and synthetic lipids alone, intermediate for BC surfactant and lowest for Infasurf (Table 1). However, around 40–50% of the material moved from the nebulizer was retained by the circuit, prior to reaching the distal end of the nasopharyngeal tubes. The relatively high residual in the nebulizer and the relatively low lung dose for Infasurf were probably secondary to higher viscosity of this clinical surfactant in comparison with the synthetic surfactant preparations.

Minimum surface tension values on the captive bubble surfactometer of SMB and BC surfactants and Infasurf were <2 mN/m before and after aerosolization, indicating persistent surface activity and integrity. Minimal surface tension for synthetic lipids alone was in the 16–19 mN/m range before and after aerosolization.

### Animal experiments

Twenty-four rabbits received anesthesia, had venous and arterial lines placed, were intubated for lung lavage, and were treated with a synthetic or clinical (Infasurf) surfactant aerosol while supported with nCPAP or mechanical ventilation (MV). Sixty minutes after surfactant administration, 2 rabbits had to be sacrificed due to worsening respiratory failure despite maximal ventilatory support: 1 in the SMB-nCPAP and 1 in the Infasurf-nCPAP group. Twenty-two rabbits completed the 2 h time course after aerosol delivery of synthetic or clinical surfactant while supported with nCPAP or MV. Twelve rabbits received aerosolized SMB (6 on nCPAP and 6 on MV), 8 received aerosolized BC surfactant (4 on nCPAP and 4 on MV), and 2 were treated with nCPAP and aerosolized Infasurf.

**Figure 1:**
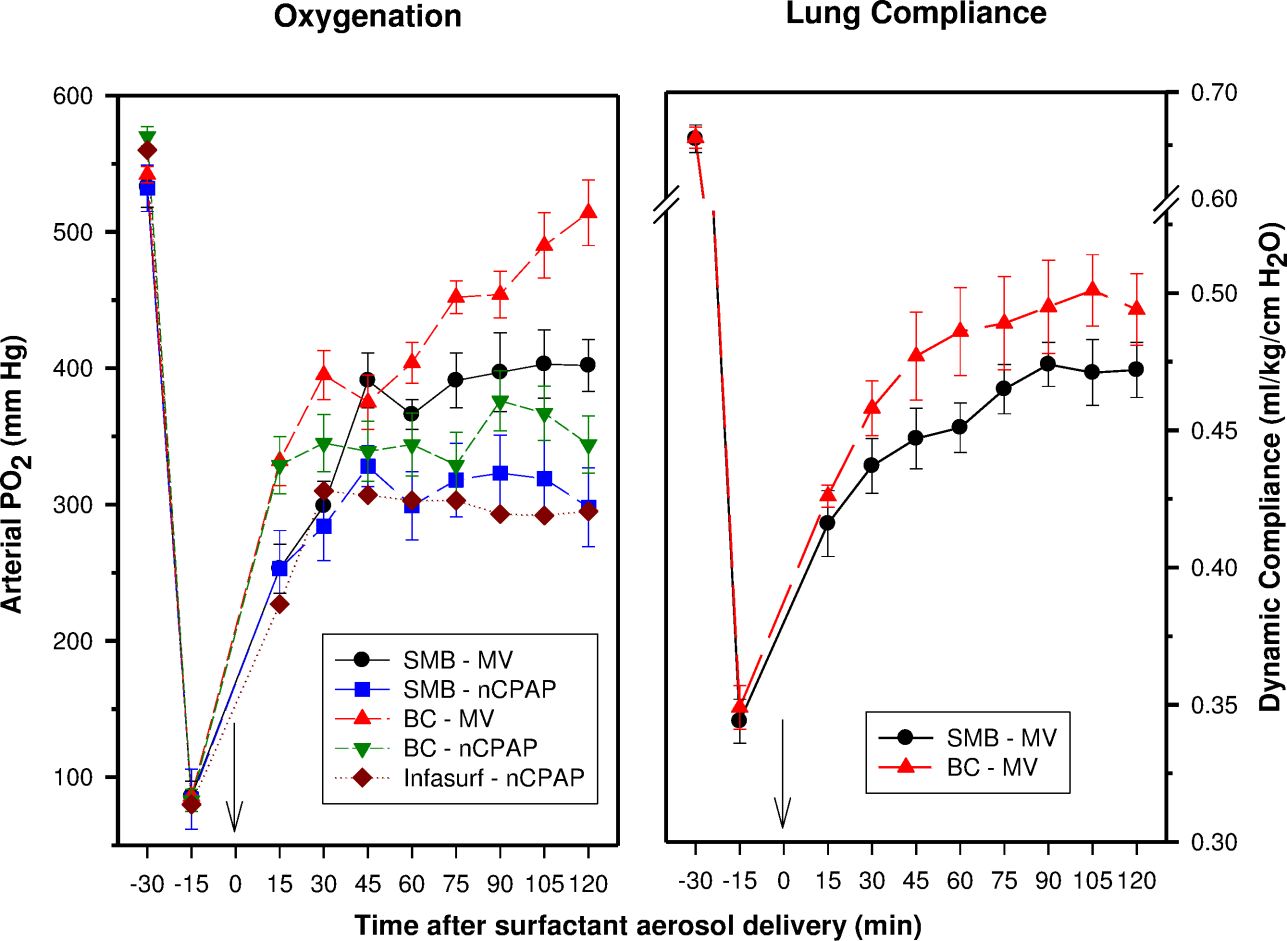
Oxygenation and dynamic compliance. Mean ± SEM of arterial PO_2_ (mm Hg) values, as a measure of oxygenation, and dynamic compliance (ml/kg/cm H_2_O) values, as a measure of lung function, in 22 saline-lavaged rabbits that received a modular dose (amount placed into the nebulizer) of 100 mg/kg of aerosolized synthetic SMB or BC surfactant or Infasurf during nasal continuous positive airway pressure (nCPAP: SMB *n* = 6, BC *n* = 4, Infasurf *n* = 2) or mechanical ventilation (MV: SMB *n* = 6, BC *n* = 4). Surfactant aerosol delivery was completed at time 0 min (arrow).

Oxygenation and lung compliance improved quickly after surfactant aerosol delivery in these rabbits. Aerosol delivery during nCPAP had a lesser effect on oxygenation than aerosol delivery during MV (Fig. 1). The differences between the nCPAP and MV subgroups were about similar for both synthetic surfactant preparations. Synthetic BC surfactant outperformed SMB surfactant with either type of ventilatory support. Infasurf aerosol delivery during nCPAP was almost as effective as SMB surfactant under these circumstances. The response to synthetic SMB and BC surfactant aerosol delivery during MV was only slightly less than the response seen after intratracheal bolus administration in previous *in vivo* experiments by our group (Walther et al., 2010; Walther et al., 2014).

### Protein and phospholipid measurements of BAL

Protein and phospholipid measurements of BAL fluid collected during the first lung lavage to induce surfactant deficiency (prior to surfactant treatment) and the first portmortem lung lavage (2 h after surfactant treatment) are shown in Table 2. Protein content of BAL fluid quadrupled in all synthetic surfactant groups during the experiments, indicating capillary-alveolar protein leakage due to acute lung injury as a result of repeated lung lavage. Phospholipid content of BAL fluid increased significantly after aerosol delivery of synthetic surfactant. Interestingly, the post-surfactant treatment phospholipid values were significantly higher (*p* < 0.02) after administration of synthetic BC than of SMB surfactant, and this difference occurred irrespective of the type of ventilatory support provided (nCPAP or MV).

**Table 2:** Protein and phospholipid content of bronchoalveolar lavages. Mean ± SEM protein and phospholipid values (µg/ml) in pre- and post-surfactant treatment bronchoalveolar lavage (BAL) fluids.

	Protein (µg/ml)		Phospholipids (µg/ml)	
Surfactant–ventilation	Pre-treatment	Post-treatment	Pre-treatment	Post-treatment
SMB-nCPAP	358 ± 96	1,796 ± 431^*^	45 ± 22	357 ± 147^*^
SMB-MV	296 ± 42	1,394 ± 259^*^	9 ± 1	437 ± 100^*^
BC-nCPAP	368 ± 58	1,731 ± 283^*^	33 ± 18	1,281 ± 172^*^^,^^**^
BC-MV	354 ± 54	1,092 ± 139^*^	34 ± 13	1,061 ± 157^*^^,^^**^

**Notes.**

* *p* < 0.001 vs pre-treatment values.

** *p* < 0.02 vs post-treatment SMB surfactant.

## Discussion

At a nominal dose (i.e., the dose loaded into the nebulizer) equivalent to that of intratracheally instilled surfactant therapy in premature infants (100 mg/kg), aerosol delivery of synthetic surfactant preparations with one SP-B peptide (Super Mini-B, SMB) or a combination of a SMB and a SP-C mimic (SP-Css ion-lock 1) (BC) in synthetic lipids (DPPC:POPC:POPG 5:3:2 weight ratio) led to a clinically relevant improvement in oxygenation in surfactant-deficient rabbits supported with nasal CPAP. *In vivo* efficacy of the experimental surfactant preparations was confirmed by comparing aerosol delivery during nCPAP support with intratracheal aerosol instillation during MV. Lung delivery of surfactant aerosol via nCPAP was less efficient than intratracheal aerosol delivery due to considerable retention of nebulized surfactant by the circuit and loss during its passage through the nasopharynx and upper airways. Increasing the lung dose by using higher surfactant dosages and/or longer delivery times of surfactant aerosol or by decreasing its viscosity by dilution with saline or possibly with water may improve this situation and needs further study. The current findings establish “proof of principle” that aerosol delivery of synthetic surfactant during nCPAP relieves surfactant deficiency in spontaneously breathing surfactant-deficient rabbits and may ultimately open up the possibility to combine nCPAP and surfactant treatment in premature infants born in low technical settings (Vidyasagar, Velaphi & Bhat, 2011).

Independent of the type of ventilatory support and, thus, the route of administration, BC surfactant (with a SP-B and a SP-C mimic) outperformed SMB surfactant (with a SP-B mimic only) in improving lung function, an observation previously made in synthetic surfactant studies in ventilated premature rabbits (Gupta et al., 2001; Almlén et al., 2010). The higher phospholipid content in BAL fluid 2 h after completion of the aerosol delivery in the BC group indicates more effective lung delivery when using BC than SMB surfactant. These findings may suggest that a synthetic surfactant preparation for aerosol delivery should contain both SP-B and SP-C mimics instead of a SP-B mimic only.

We routinely use synthetic lipids alone as a negative control, and a clinical surfactant, such as Infasurf, as a positive control in our *in vitro* and *in vivo* experiments. In this study we first ran our tests with the nebulizer with synthetic lipids alone, SMB surfactant, and Infasurf. Because intratracheal bolus instillation of synthetic lipids only starts to improve oxygenation and lung compliance after a lag time which surpasses the duration of the experiments, we refrained from their use in the aerosol experiments. In contrast with SMB-surfactant, Infasurf repeatedly clogged the system due to its higher viscosity and may have to be diluted in future aerosol experiments. Based on the results of the initial animal experiments with SMB-surfactant and our recent experience with the development of various highly surface active SP-C mimics (RH Notter, AJ Waring, FJ Walther, LM Gordon, and Z Wang, 2012, unpublished data), we decided to expand our study by testing a synthetic surfactant preparation with a SP-B and a SP-C mimic. We chose the SP-Css ion-lock 1 peptide as it is a stable SP-C mimic with enhanced *in vitro* and *in vivo* surfactant activities. Addition of SP-Css ion-lock 1 to SMB led to a more complete surfactant and nicely demonstrated the additive effects of a SP-C mimic.

There is clearly an unmet demand in clinical neonatology for an aerosolized surfactant preparation for inhalation therapy in spontaneously breathing newborn infants supported with nCPAP (Vidyasagar, Velaphi & Bhat, 2011). The equivocal results with various first generation liquid surfactant preparations have hampered progress in this field, but the development of new liquid and dry powder nebulizers and advances in synthetic surfactant design will surely provide new impetus. Several research groups have explored the potential of dry powder aerosolization of a recombinant SP-C surfactant (Venticute^®^, Nycomed GmbH, Konstanz, Germany) in mice, rabbits and premature lambs and shown its potential to deliver high doses comparable to those used for intratracheal bolus instillation (Ruppert et al., 2010; Rahmel et al., 2012; Pohlmann et al., 2013). However, surface activity of the recombinant SP-C surfactant has recently come under attack after disappointing results in clinical studies of patients with acute lung injury (Spragg et al., 2011). A recent study (Lampland et al., 2014) in spontaneously breathing newborn pigs with acute lung injury showed similar improvements in short-term survival and pulmonary gas exchange with aerosolized and intratracheally instilled liquid KL_4_ surfactant (Surfaxin^®^, Discovery Laboratories Inc., Warrington, PA, USA). However, the oxygenation response in the spontaneously breathing newborn pigs was rather limited with PaO_2_ values ∼200 mm Hg, indicating the limited surface activity of KL_4_-based surfactant mixtures (Walther et al., 1998). Altogether, the above mentioned studies indicate the potential of aerosolized synthetic surfactant preparations with a single SP-B or SP-C mimic. Our current study does not only confirm these findings, but demonstrates the far greater potential of synthetic lung surfactant with a combination of highly active second generation SP-B and SP-C mimics.

## Conclusions

Aerosol delivery of synthetic lung surfactant with a combination of highly active second generation SP-B and SP-C mimics was effective as a therapeutic approach towards relieving surfactant deficiency in spontaneously breathing rabbits supported with nCPAP. However, to obtain similar results with nCPAP as with intratracheal instillation during mechanical ventilation, further optimization of the delivery of these second generation surfactants, e.g., by increasing dosages, delivery times and reducing viscosity is indicated.
